# Dual-controlled optogenetic system for the rapid down-regulation of protein levels in mammalian cells

**DOI:** 10.1038/s41598-018-32929-7

**Published:** 2018-10-09

**Authors:** Julia Baaske, Patrick Gonschorek, Raphael Engesser, Alazne Dominguez-Monedero, Katrin Raute, Patrick Fischbach, Konrad Müller, Elise Cachat, Wolfgang W. A. Schamel, Susana Minguet, Jamie A. Davies, Jens Timmer, Wilfried Weber, Matias D. Zurbriggen

**Affiliations:** 1 Faculty of Biology, University of Freiburg, Freiburg, Germany; 2 BIOSS - Centre for Biological Signalling Studies, University of Freiburg, Freiburg, Germany; 3 Institute of Physics, University of Freiburg, Freiburg, Germany; 4 Deanery of Biomedical Sciences, University of Edinburgh, Edinburgh, UK; 5 SGBM - Spemann Graduate School of Biology and Medicine, University of Freiburg, Freiburg, Germany; 6 Institute of Synthetic Biology, University of Düsseldorf and CEPLAS, Düsseldorf, Germany; 7 Center for Chronic Immunodeficiency, Medical Center, Faculty of Medicine, University of Freiburg, Freiburg, Germany; 8 Present Address: Institute of Chemical Sciences and Engineering, School of Basic Sciences, Ecole Polytechnique Fédérale de Lausanne (EPFL), Lausanne, CH-1015 Switzerland; 9 Present Address: Novartis Pharma AG, Basel, CH-4002 Switzerland

## Abstract

Optogenetic switches are emerging molecular tools for studying cellular processes as they offer higher spatiotemporal and quantitative precision than classical, chemical-based switches. Light-controllable gene expression systems designed to upregulate protein expression levels meanwhile show performances superior to their chemical-based counterparts. However, systems to reduce protein levels with similar efficiency are lagging behind. Here, we present a novel two-component, blue light-responsive optogenetic OFF switch (‘Blue-OFF’), which enables a rapid and quantitative down-regulation of a protein upon illumination. Blue-OFF combines the first light responsive repressor KRAB-EL222 with the protein degradation module B-LID (blue light-inducible degradation domain) to simultaneously control gene expression and protein stability with a single wavelength. Blue-OFF thus outperforms current optogenetic systems for controlling protein levels. The system is described by a mathematical model which aids in the choice of experimental conditions such as light intensity and illumination regime to obtain the desired outcome. This approach represents an advancement of dual-controlled optogenetic systems in which multiple photosensory modules operate synergistically. As exemplified here for the control of apoptosis in mammalian cell culture, the approach opens up novel perspectives in fundamental research and applications such as tissue engineering.

## Introduction

A common approach to study the function of a protein of interest in mammalian cells is to artificially manipulate its expression level. This approach is versatile, as it can be applied to most types of proteins, and simple, since no regulation mechanisms of the endogenous proteins need to be known or modulated.

Initially, chemical-based switches were used to manipulate expression levels by controlling transcription. Such systems are based on transcriptional activators or repressors, which alter their conformation and hence their target DNA-binding affinity upon interaction with specific small molecules. However, chemical-based switches have many limitations such as potential toxic or off-target effects of the regulatory small molecule, its poor or unpredictable diffusion through tissues and the difficulty of removing it from cells, tissues or organisms^1^. Optogenetic systems have the potential to overcome these limitations, creating great interest in implementing them in animal cell systems in culture and *in vivo*^2,3^. As optogenetic systems offer almost unlimited spatiotemporal resolution, dozens of switches have recently been implemented for the control of a wide range of intracellular processes including protein localization^4^, activity^5^ and stability^6,7^, multi-wavelength gene-expression control^8–11^ and organelle motility^12^.

Optogenetic switches that control cellular protein abundance have in the last years shown much relevance and utility in biological research^2^. Most systems developed to date are based on the light-regulated induction of gene expression, some of which are reversible by illumination^8^, therefore allowing high temporal and quantitative control. Systems to reduce protein levels are, however, not as established. Currently, only few systems are capable of down-regulating protein levels^6,13^. However, none of the existing systems actively represses transcription which would contribute to an efficient and quantitative reduction of protein levels. To address this limitation, we envisioned that an optogenetic system that actively represses promoter activity and simultaneously targets protein stability would result in superior reduction of cellular protein levels in terms of rate and quantitative control. For this purpose, we have developed a novel dual-controlled optogenetic system (‘Blue-OFF’) that combines transcriptional repression with regulation of protein stability, upon illumination with a single wavelength. The Blue-OFF system consists of two blue light-responsive protein modules: a novel, light-responsive repressor, KRAB-EL222, and the protein degradation module B-LID^6,9^. Both components utilize light-oxygen-voltage (LOV) domains, which react to blue light illumination using a flavin mononucleotide (FMN) as chromophore. Blue light illumination induces an adduct formation between FMN and a cysteine in the LOV domain, which triggers a conformational change in the protein, changing its effector function^14,15^.

EL222 is a photosensitive transcription factor from the bacterium *Erythrobacter litoralis*. This transcription factor consists of a light sensitive LOV domain and a helix-turn-helix (HTH) DNA-binding domain, which can mediate light-induced transcription activation. In the dark, the LOV domain binds the HTH domain, precluding dimerization of the transcription factor and therefore no specific binding to a cognate DNA-sequence takes place. Blue light illumination disrupts the inhibitory LOV-HTH interactions and allows EL222 to homodimerize and bind specifically to the DNA sequence (C120). This interaction spontaneously reverses in the dark rendering EL222 inactive (τ ~ 11 s at 37 °C)^16–18^. EL222 has already been adapted for light controllable transcriptional activation in mammalian cells by fusing it to a virus-derived transactivator domain^9^. We demonstrate for the first time its use as light-inducible transcriptional repressor by fusing it to the KRAB transrepressor domain to inhibit transcription from a constitutive promoter^19^.

The B-LID module incorporates the LOV2 domain from *Avena sativa* phototropin 1 (AsLOV2). Illumination of AsLOV2 leads to an unwinding of the C-terminal Jα helix that is bound to the LOV core domain in the dark^6^. This structural change reverses spontaneously in darkness (τ ~ 80 s at 22 °C)^6^. This mechanism can be exploited by integrating small peptide tags in the C-terminal Jα helix which then are structurally hidden in the dark and are only exposed upon illumination. For the development of the B-LID module, the peptide sequence RRRG was fused to the Jα helix of AsLOV2 leading to the light inducible proteasome-mediated degradation of a linked protein^6,7,15^.

**Figure 1:**
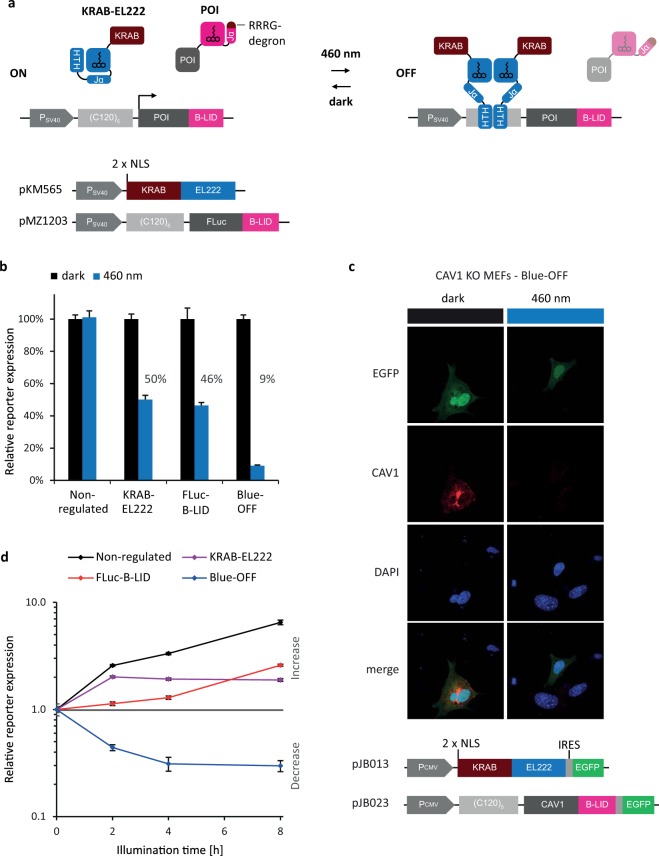
Design and validation of the Blue-OFF system. (**a**) Mode of function and constructs. Expression of the reporter protein FLuc-B-LID is placed under the control of a SV40 promoter followed by five copies of the EL222-binding sequence, (C120)_5_. The photosensitive transcription factor EL222 is fused to an inhibitory KRAB domain and to two nuclear localization sequences (NLS). In the dark, KRAB-EL222 cannot bind to (C120)_5_. Upon blue light illumination, KRAB-EL222 dimerizes and binds to (C120)_5_ sequence inhibiting transcription. FLuc is fused to a B-LID module: in the dark the degradation peptide (RRRG) is docked to the LOV domain and thus covered. Blue light illumination exposes the peptide and leads subsequently to proteasome-mediated protein degradation. (**b**) Validation of the combined transcriptional and post-translational regulation. HEK-293T cells were transfected transiently with either no blue light-sensitive regulation module (Non-regulated: pWW43 + pMZ1210), single regulation modules (KRAB-EL222 only: pKM565 + pMZ1210; or FLuc-B-LID only: pWW43 + pMZ1203) or both modules together for the Blue-OFF system (pKM565 + pMZ1203). The cells were kept either in darkness for 24 h (black bars) or for 16 h in the dark conditions and then illuminated with 460 nm light for 8 h (blue bars). FLuc levels shown here are normalized to their dark control. (**c**) Constructs of the CAV1-Blue-OFF system. In darkness CAV1 accumulates whereas under blue light illumination active repression of transcription and degradation leads to a net decrease of CAV1 levels. CAV1 knock out (KO) primary embryonic fibroblast cells were transfected with KRAB-EL222 and CAV1-B-LID (pJB013 and pJB023, respectively). After transfection cells were illuminated with 2 µmol m^−2^ s^−1^ of 460 nm light for 16 h. After fixation and permeabilization, cells were stained with an anti-CAV1 antibody followed by an AlexaFluor546-labelled secondary antibody and nuclei were counterstained with DAPI. Cells were imaged by confocal microscopy. (**d**) Kinetics of the blue light regulation systems. HEK-293T cells were transfected as before and incubated in darkness for 16 h. Cells were then illuminated for 0, 2, 4 and 8 h with blue light. FLuc levels were measured at the indicated time points and are represented normalized to the values obtained after 16 h darkness. In b and c, data are means of four independent replicates and error bars indicate standard deviation of the mean.

We present the combination of both modules in a single optogenetic system, which allows accurate control of protein production simultaneously on transcriptional and post-translational levels (Fig. 1a). This combinatorial approach enables a stronger, faster and longer-lasting reduction of cellular protein levels compared to the single modules, demonstrating the effectiveness of integrating repression on a transcriptional as well as a post-translational level. As a proof of principle, we have demonstrated the functionality of Blue-OFF with the reporter protein firefly luciferase and the mouse protein Caveolin-1 (CAV1). We show here that the Blue-OFF repression system can efficiently reduce protein expression levels, in different mammalian cell lines. Moreover, we developed a mathematical model to describe the activity of the Blue-OFF system which can hence be used for the experimental design by guiding the choice of irradiation conditions for obtaining desired repression levels. In line with these results, we further showed the applicability of the system for the optogenetic control of programmed cell death in mammalian cells by combining a drug-controlled caspase with the Blue-OFF system.

We present the first optogenetic repression system based on multiple photoreceptor modules that combines active repression with the control of protein stability and establish its use in a synergistic system. Taken together, our data highlight the advantages and strengths of this novel tool to complement the optogenetic toolbox.

## Results

### Design of a high-performance repression system by combining optogenetic modules

The optogenetic repressor module KRAB-EL222 was constructed by fusing a repressive KRAB domain, derived from the human *kox-1* gene^19^, and two nuclear localization signals (NLS) to the N-terminus of EL222. The KRAB-EL222 module was cloned into an SV40 promoter-driven mammalian expression vector (pKM565) (Fig. 1a). As a light-regulated protein degradation system, we chose the B-LID system^6^. The functionality of both modules was assayed using a firefly luciferase (FLuc) reporter (pMZ1203), constructed to combine transcriptional and post-translational regulation (Fig. 1a): Transcriptional regulation was achieved by placing the FLuc under the control of a constitutive SV40 promoter followed by five copies of the EL222-binding sequence, (C120)_5_, for binding of KRAB-EL222. Protein stability control was provided by fusing the B-LID degradation module C-terminally to FLuc. We first characterized the functionality of each module independently. The B-LID system was tested by replacing the EL222-KRAB module with the E-KRAB (pWW43) variant that cannot bind to (C120)_5_. To test the KRAB-EL222 module independently, a reporter similar to pMZ1203 but lacking the RRRG degradation sequence (pMZ1210) was constructed. HEK-293T cells were transfected with the indicated plasmids in a 1:1 (w:w) ratio, incubated in the dark for 16 h, and illuminated for 8 h with 20 µmol m^−2^ s^−1^ of 460 nm light (Fig. 1b). Cells transfected with the two control plasmids showed no difference in luciferase expression between illuminated and non-illuminated cells (Fig. 1b, ‘Non-regulated’). Cells transfected with only the KRAB-EL222 system showed a 50% repression in cells illuminated for 8 h, compared to those kept in the dark, demonstrating the functionality of this new photosensitive repressor. Additionally, we engineered and tested a set of variants of this module, none of which showed any better repressive behavior (Fig. S1). Cells transfected with only the B-LID system showed 46% of the expression in non-illuminated cells. The dual-regulated system exhibited stronger repression, with only ~10% of the control levels of protein remaining. The level of down-regulation achieved is even stronger than a pure multiplicative combination of the two single modules. This result is due to nonlinearities in the system and shows a synergistic effect of the two optogenetic modules (Fig. 1b, ‘Blue-OFF’). To extend the applicability of the dual-controlled Blue-OFF repression system we investigated its ability to down-regulate proteins other than reporters, e.g. Caveolin-1 (CAV1). CAV1 is the major component of endocytic caveolae plasma membrane invaginations and plays a critical role in normal tissue architecture and tumor progression^20,21^. In order to test the system, we employed the Blue-OFF system for controlling CAV1 levels (Fig. 1c). The plasmids contained EGFP as reporter to monitor transfected cells. The system was transfected in CAV1 knock-out (KO) mouse embryonic fibroblasts (MEFs). Cells were illuminated for 16 h with 2 µmol m^−2^ s^−1^ of 460 nm. CAV1 expression levels were evaluated using immunofluorescence microscopy (Figs 1c, S2). As expected, CAV1-KO cells transfected with Blue-OFF and kept in darkness showed high expression of CAV1, whereas after blue light illumination, CAV1 levels significantly decreased but not co-expressed EGFP signals.

Following these results, we set out to characterize the kinetics of repression, to gain a better insight into the contribution of transcriptional and posttranslational regulation on performance of the dual-regulated Blue-OFF system. Cells were incubated for 16 h in the dark followed by 0, 2, 4 or 8 h of illumination prior to the determination of luciferase activity (Fig. 1d). The non-regulated, blue light-insensitive control system showed ongoing protein accumulation unaffected by illumination (black line). In contrast, protein accumulation was halted by controlling only transcription using KRAB-EL222 (purple line). The repression of transcription first became apparent on the protein level 2 h after illumination, when no further increase, but a slow decrease of protein accumulation was observed. The 2 h delay is likely the result of ongoing translation from already synthesized mRNA during this time. In contrast, posttranslational control via protein degradation with the B-LID system showed a stronger and more immediate effect than transcriptional regulation (red line). The dual-regulated Blue-OFF system integrated the rapid, but temporally limited degradation effect of the B-LID system, with a delayed but persistent repressive effect of KRAB-EL222 to achieve a faster, stronger and longer-lasting repression (blue line). Furthermore, in contrast to the systems using a single optogenetic module, only the dual-controlled system achieved an absolute decrease in cellular protein levels after starting illumination. Our data demonstrates that the combination of transcriptional and posttranslational regulation in the Blue-OFF system results in a superior, light-induced down-regulation of a protein of interest.

### Reversibility and versatility of the dual-controlled optogenetic Blue-OFF system

**Figure 2:**
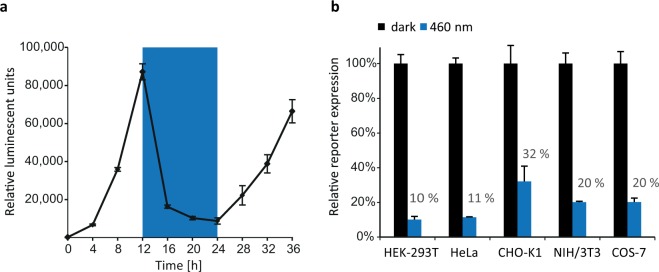
Reversibility and versatility of the Blue-OFF system. (**a**) HEK-293T cells were transfected with KRAB-EL222 (pKM565) and FLuc-B-LID (pMZ1203).and kept in darkness for 12 h followed by 12 h blue light illumination and again 12 h darkness. FLuc levels were measured every 2 h. (**b**) Blue-OFF characterization using different mammalian cell lines. The indicated cell lines were transfected with KRAB-EL222 and FLuc-B-LID. Cells were kept in darkness for 16 h followed by 8 h of 460 nm blue light illumination. FLuc levels were determined at the final time point. To correct for different transfection efficiencies, the expression data were normalized to co-transfected constitutively expressed Renilla luciferase (RLuc). In a and b, data are means of four independent replicates and error bars indicate standard deviation of the mean.

A key advantage of light as an inducer is its high temporal precision and the reversibility of its application to a given biological system^22^. To evaluate reversible control of protein expression, HEK-293T cells expressing the Blue-OFF system were kept in darkness for 12 h, followed by a cycle of 12 h blue light illumination and 12 h darkness. Blue light illumination resulted in a 90% reduction of the protein level reached in darkness. Another 12 h of darkness allowed the protein levels to recover, demonstrating that the Blue-OFF system can control protein expression in a reversible manner (Fig. 2a).

To validate versatility, the Blue-OFF system was implemented in different mammalian cell lines. To this end, we expressed the system transiently in human embryonic kidney cells (HEK-293T), human cervical cancer cells (HeLa), chinese hamster ovary cells (CHO-K1), mouse embryonic fibroblasts (NIH/3T3) and monkey fibroblast-like cells (COS-7). Cells were kept in darkness for 16 h, followed by 8 h of blue light illumination. Blue light illumination resulted in a 70% to 90% decrease of reporter protein expression among the various cell lines, suggesting a high versatility and cross-species applicability of the Blue-OFF system (Fig. 2b).

### Development of a quantitative model to describe the activity of the Blue-OFF system

To further characterize the Blue-OFF system and its contributing modules, we developed a mathematical model based on ordinary differential equations (ODE) describing the time evolution of the concentrations of the involved substances. The model is parameterized using quantitative data on the time course and on the response to light intensity.

1 $$\frac{d[FLu{c}_{off}](t)}{dt}=-\,{k}_{deg,const}[FLu{c}_{off}]+{k}_{translate}[FLu{c}_{mRNA}]-{k}_{on}I(t)[FLu{c}_{off}]+{k}_{off}[FLu{c}_{on}]$$

2 $$\frac{d[FLu{c}_{on}](t)}{dt}=-\,{k}_{deg,const}[FLu{c}_{on}]-\frac{{k}_{deg,ind}[FLu{c}_{on}]}{{K}_{m,deg}+[FLu{c}_{on}]}+{k}_{on}I(t)[FLu{c}_{off}]-{k}_{off}[FLu{c}_{on}]$$

3 $$\frac{d[KRA{B}_{off}](t)}{dt}=-\,{k}_{on}I(t)[KRA{B}_{off}]+{k}_{off}[KRA{B}_{on}]$$

4 $$\frac{d[KRA{B}_{on}](t)}{dt}=+{k}_{on}I(t)[KRA{B}_{off}]-{k}_{off}[KRA{B}_{on}]$$

5 $$\frac{d[FLu{c}_{mRNA}](t)}{dt}=\frac{{k}_{transcript}}{1+{k}_{inh,KRAB}{[KRA{B}_{on}]}^{2}}-{k}_{deg,mRNA}[FLu{c}_{mRNA}]$$

The Blue-OFF system is based on the light-induced conformational change of two proteins. On the one hand, the FLuc-B-LID switch exists in two conformations: FLuc_off_ and FLuc_on_. FLuc_off_ is the form present in the dark with an inactive B-LID domain which is translated from FLuc_mRNA_ and degrades at the rate k_deg,const_. By illumination with blue light with the intensity I(t), the protein changes its conformation to FLuc_on_ and is available for degradation via the proteasome. The parameter K_m,deg_ describes saturation of the proteasome-dependent protein-degradation machinery. On the other hand, the light-inducible transcriptional repressor EL222-KRAB is in an inactive form in the dark, KRAB_off_. Upon illumination with blue light the conformation is changed to KRAB_on_, which represses the transcription of the FLuc_mRNA_. The FLuc_mRNA_ is produced with the constitutive transcription rate k_transcript_ in the absence of EL222-KRAB. A detailed derivation of the model equations can be found in the Supplementary Information.

**Figure 3:**
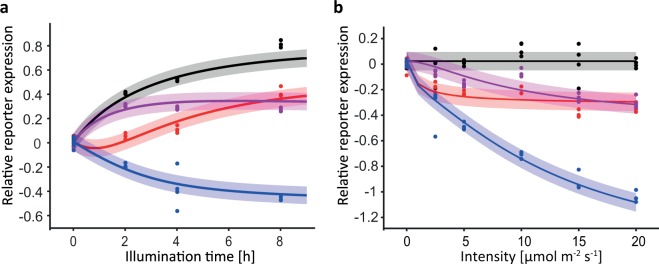
Quantitative characterization of the behavior of the Blue-OFF system to calibrate the mathematical model. The model was calibrated using kinetic (**a**) and intensity dose response data (**b**). For the dose response HEK-293T cells were transfected with the Blue-OFF system. The cells were kept in darkness for 16 h after transfection and subsequently illuminated for 8 h with 0, 2.5, 5, 10, 15 and 20 µmol m^−2^ s^−1^ of 460 nm light. Shown is the relative reporter expression on a logarithmic scale. The dots indicate the experimental data points and the solid lines show the model simulation for the optimal parameter set. The shaded error bands are estimated by using an error model assuming a log-normally distributed error.

The model was calibrated by using the measured kinetics (Figs 1d and 3a) and light intensity ‘dose’-response data (Fig. 3b). The parameters were estimated by maximizing the likelihood function. The resulting fit is shown in Fig. 3. The model can explain the measured data, including the high synergistic repression in the combined system. An analysis of the calibrated model suggests as reason the saturation in the process of the B-LID induced protein degradation. In cells transfected only with the B-LID system the FLuc degradation is saturated whereas for the combined system the FLuc concentration is lower therefore not reaching saturation in its degradation. To assess the uncertainties of the estimated parameters, we calculated the profile likelihood of each parameter^23^. The detailed fitting process, estimated parameter values and their 95% confidence intervals are shown in the Supplementary Information. The parameter estimation and the profile likelihood analysis were performed with the Data2Dynamics framework^24^.

### Implementation of the mathematical model to predict experimental outcomes

**Figure 4:**
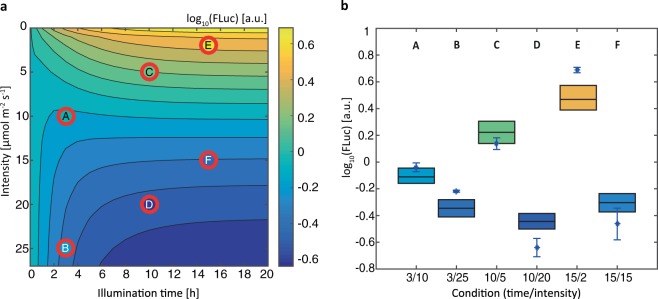
Model-aided prediction of protein expression levels using the Blue-OFF system. (**a**) Simulated reporter expression for different light intensities and illumination times for characterizing the system. To validate if the model can be used to determine experimental conditions to obtain a desired protein level six combinations with different intensities and illumination durations were measured and compared to the model predictions (red circles). The colored filling in the red circles indicates the experimentally determined expression levels. (**b**) The means of four determinations for each combination are denoted with blue stars and the error bars are showing the standard error of the mean. The colored boxes show the 95% prediction confidence interval calculated by analyzing the prediction profile likelihood.

Next, we aimed to control the level of repression in a predictable manner by adjusting the illumination time and intensity using the calibrated mathematical model to predict the performance and operating range of the system. Figure 4a shows the predicted expression level of FLuc for different light intensities and illumination times. This map helps identifying the illumination conditions needed to obtain a desired reporter expression level.

Several experimental conditions were selected (A-F in Fig. 4a) and the reporter protein levels were compared to the values predicted from the mathematical model to test the applicability of the model to achieve a desired expression level by choosing the illumination time and light intensity (Fig. 4b). The uncertainties of the estimated model parameters lead to an uncertainty of the model prediction. To quantify this uncertainty in terms of prediction confidence intervals, we analyzed the prediction profiles likelihood^25^ (Fig. 4b, colored boxes). The predicted expression levels (colored boxes) and the experimentally obtained expression levels (individual blue stars) show a strong correlation. These results indicate the applicability and usefulness of the model to determine conditions for experimental setups to achieve specific repression levels. Moreover, this shows the highly tunable repression levels of the Blue-OFF system by varying light intensity or illumination time.

### Controlling programmed cell death using the Blue-OFF system

**Figure 5:**
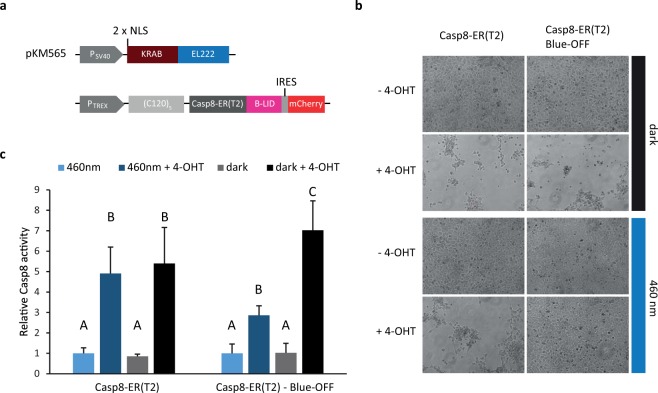
Application of the Blue-OFF system for the control of programmed cell death. (**a**) Constructs of the Casp8-ER(T2)-Blue-OFF system. (**b**,**c**) Optogenetic control of programmed cell death in HEK-293 cells transfected with the Casp8-ER(T2)-Blue-OFF system. (**b**) HEK-293 cells transfected with the Casp8-ER(T2) or the Casp8-ER(T2)-Blue-OFF systems form a uniform monolayer under blue light exposure or in darkness in the absence of 4-OHT. Induction of caspase 8 activity upon 4-OHT addition leads to cell death in darkness (for Casp8-ER(T2) and Casp8-ER(T2)-Blue-OFF) and under blue light exposure for Casp8-ER(T2), whereas cells transfected with the Casp8-ER(T2)-Blue-OFF system show a higher survival rate under blue-light conditions, thus building a uniform cell monolayer. (**c**) Quantification of caspase8 activity. The figure shows the caspase 8 activity of HEK-293 cells transfected with the Casp8-ER(T2) system (left) or the light-regulated Casp8-ER(T2)-Blue-OFF system (right), in the presence and absence of 4-OHT and blue light. Values are mean of three independent experiments and error bars indicate standard deviation of the mean. Statistical significance between the tested conditions for each system is indicated with uppercase letters above each bar, where “A” significantly differs from “B”, “B” from “C”. One-way analysis of variance (ANOVA), *P* < 0.005.

Finally, we set out to implement the Blue-OFF system to achieve light control over apoptosis in mammalian cells. For this purpose, we customized the tamoxifen inducible apoptosis-inducing caspase protein (Casp8-ER(T2)), previously described by Cachat *et al*.^26^. Caspase 8 is predominantly present as an inactive monomer, but upon addition of 4-hydroxytamoxifen (4-OHT) the ER(T2) domain brings about dimerization and activation, triggering apoptosis^27^. We integrated this module into the Blue-OFF system for blue-light control of caspase8 stability (Casp8-ER(T2)-Blue-OFF) (Fig. 5a). HEK-293 cells transfected with Casp8-ER(T2) showed significant apoptosis upon induction with 4-OHT both in the dark or under blue light, whereas cells transfected with the Casp8-ER(T2)-Blue-OFF system were able to grow and form a monolayer under blue light (Fig. 5b).

Subsequently, a caspase 8 activity assay was performed in order to correlate the protective effect of the dual Blue-OFF system on cell survival with reduced activity of caspase 8. After addition of 4-OHT, a 7-fold increase in caspase 8 activity was observed (Fig. 5c) in cells transfected with the Casp8-ER(T2)-Blue-OFF system when kept in the dark compared to transfected cells without addition of 4-OHT. When illuminated with blue light a 60% reduction in caspase 8 activity was observed (Fig. 5c), which is in line with the reduced cell death (Fig. 5b). As expected, blue light had no effect on caspase 8 activity in 4-OHT induced control cells transfected with Casp8-ER(T2). These results indicate that the Blue-OFF system can efficiently be used for the optogenetic control of programmed cell death in mammalian cells and opens up the possibility for the selective induction of apoptosis in specific cells by local illumination that is unachievable with diffusible drugs.

## Discussion

The recent development of light-regulated synthetic molecular switches has considerably contributed to a better insight into the functions and characteristics of proteins in regulatory networks (www.optobase.org)^28^. A common function of these switches is to upregulate protein abundance in biological systems, their subcellular localization or their activity^29^. While there is a broad set of optogenetic tools available to upregulate protein expression, only few systems are available to reduce protein levels. Despite successful results in various experimental designs, these down-regulation approaches are not as efficient in terms of absolute reduction of protein levels.

To improve the efficiency of transgene expression control, transcription-translation networks have been developed. These dual approaches enable tighter control of protein expression, however until recently mainly relied on chemical switches^13,30–33^.

For this purpose, we engineered a dual-controlled optogenetic switch, Blue-OFF, that confers rapid and sustained, blue light sensitive down-regulation of protein expression levels by simultaneously targeting gene repression and protein stability. The novel blue light-responsive repressor KRAB-EL222 was combined with the protein degradation module B-LID constituting a system that can be regulated upon illumination with a single wavelength.

By combining both levels of regulation the Blue-OFF system outperforms existing down-regulation systems.

Efficient down-regulation of protein expression is instrumental in answering many biological questions. Biological systems are highly dynamic, thus, reversibility is a key feature of synthetic molecular switches to better understand biological processes. Therefore, light emerges as optimal superior inducer, since it can be applied and withdrawn from biological systems in a fully reversible manner. Here, we showed how protein down-regulation by the Blue-OFF system is fully reversible. Moreover, we were able to validate the functionality of the system in different cell types, which proves its broad applicability.

To quantitatively understand the underlying processes in light-inducible gene repression and protein degradation, we developed a quantitative mathematical model that was parameterized with the experimental data. Using this model, it is possible to set the experimental parameters (light intensity and illumination regime) for tuning the Blue-OFF for desired applications. Blue-OFF can, furthermore, easily be combined with other systems by placing the target site of the repressor modules KRAB-EL222 behind any endogenous or synthetic promoter and by fusing B-LID to any protein of interest. The Blue-OFF was successfully applied for the control of programmed cell death which opens up novel perspectives for creating cellular patterns with high spatiotemporal resolution. In the frame of the current development of strategies for engineering multicellular systems, the Blue-OFF system could contribute to synthetic tissue engineering approaches and the generation of complex 3D structures.

In conclusion, we have shown that the novel Blue-OFF optogenetic approach for controlling protein levels, acting simultaneously on transcriptional and post-translational levels, leads to a fast and strong reduction of the net level of the protein of interest. In addition, a model-based quantitative characterization of the system kinetics enables the rational adjustment of parameters to achieve desired repression levels. The strong repressive effect together with the predictive properties of the system constitute a powerful and versatile tool. For the future, we envision that this system will be used to answer fundamental biological questions and boost applications such as in tissue engineering.

## Methods

### Plasmids

The design and the construction of the expression vectors are described in Tables S2, S3.

### Cell culture and transfections

Chinese hamster ovary cells (CHO-K1) were cultivated in HTS medium (Cell Culture Technologies) supplemented with 10% tetracycline-free fetal calf serum (FCS, PAN, cat. no.: P30–3602, lot no.: P101003TC), 2 mM L-glutamine (Sigma), 100 U mL^−1^ penicillin and 0.1 mg mL^−1^ streptomycin (PAN). Mouse embryonic fibroblast cells (NIH/3T3), human embryonic kidney cells (HEK-293T), African green monkey fibroblast-like cells (COS-7), and human epithelioid cervix carcinoma cells (HeLa) were maintained in Dulbecco’s modified Eagle’s medium (DMEM, PAN, cat. no. P04–03550) supplemented with 10% FCS (FCS, PAN, cat. no.: P30-3306, lot no.: P140204), 100 U mL^−1^ penicillin and 0.1 mg mL^−1^ streptomycin (PAN) at 37 °C with 5% CO_2_. For transfection, 30,000-75,000 cells per well of a 24-well plate were transfected using polyethylenimine (PEI, linear, MW: 25 kDa, Polyscience) as describes elsewhere^34^. Unless otherwise indicated, cells were transfected with constructs comprising the light responsive repressor KRAB-EL222 (pKM565), FLuc-B-LID (pMZ1203) and as a control CMV driven Renilla Luciferase (RLuc) at a ratio of 20:20:1 (w:w:w), respectively.

Immortalized mouse embryonic fibroblasts (MEFs) were obtained from Caveolin 1-deficient mice (B6.Cg-CAV1tm1mls/J^35^). Immortalization was induced by the simian virus 40 large T antigen (SV40-Tag). Cells were cultured in DMEM (1×) + GlutMAX^TM^ (Gibco, cat. no.: 61965–026) supplemented with 10% fetal bovine serum (Gibco, cat. no.: 10270–106, lot no.: 42F9251K), 50 U mL^−1^ penicillin and 0.05 mg mL^−1^ streptomycin (Gibco, cat. no.: 15140–122) and 50 µM β-mercaptoethanol (Sigma). Transfections for MEFs were done using TransitX2 (Mirus, cat. no.: MIR6000). 70.000 cells were seeded in 12-well plates and transfected using 1 µg of DNA/3 µl TransitX2.

Human embryonic kidney (HEK-293) cells were maintained in DMEM (Gibco, cat. no.: 41966) supplemented with 10% fetal bovine serum (Biosera, cat. no.: FB1090/500, lot no.: 013BS145) in a humidified incubator at 37 °C and 5% CO_2_. HEK-293 cells were harvested by trypsinization 24 h prior to transfection, and seeded at a density of 80.000 cells in 500 µl of complete medium per well on 24 well plates. Cells were transfected with lipofectamine 3000 (Invitrogen, cat. no.: L3000–008). Unless otherwise indicated cells were transfected with pTREX-BLID-mCherry-2A-myrcasp8-ER(T2), pKM565 or pTREX-myrCasp8-ER(T2)-IRES-mCherry. After 24 hours, the medium was replaced by fresh growth medium.

### Light induction

Cells were kept in darkness or were illuminated with 460 nm light for the indicated time periods at a photon flux density of 20 µmol m^−2^ s^−1^, unless indicated otherwise. Illumination was performed with light boxes similar to^36^ with LED panels emitting at 460 nm (LED Engin, cat. no.: LZ1-10B202-0000). All cell-handling involving the blue-light inducible systems was done under 628 nm light which does not affect the light-sensitive systems described here.

### Reporter gene assay

Luciferase expression was quantified by lysing cells on ice with 250 µl luciferase lysis buffer (25 mM Tris/HCl, pH 7.8, 1% Triton X-100, 15 mM MgSO_4_, 4 mM ethylene glycol tetraacetic acid (EGTA), 1 mM DTT) per well on ice for 15 min. 80 µl lysate was transferred to Costar® 96-well flat-bottom white plates (Corning Incorporated, Germany). Firefly and Renilla luciferase luminescence was directly monitored using either a Synergy 4 multimode microplate reader (BioTek Instruments Inc., Winooski, VT) or an Infinite 200Pro microplate reader (Tecan, Switzerland) after addition of 20 μl of either firefly luciferase substrate (20 mM Tricine, 2.67 mM MgSO_4_, 0.1 mM EDTA, 33.3 mM DTT, 0.52 mM ATP, 0.27 mM Acetyl-CoA, 5 mM NaOH, 264 µM MgCO_3_, 0.47 mM luciferin) or Renilla luciferase substrate (472 μM coelenterazine stock solution in methanol; diluted directly before use, 1:15 in PBS).

### Immunofluorescence microscopy

Cells were fixed in 4% paraformaldehyde for 15 min at room temperature and washed three times in PBS, permeabilized in 0.5% Triton X-100 for 15 min at room temperature, rewashed in PBS and blocked in PBS with1% BSA for 30 min at room temperature. Subsequently, cells were incubated overnight at 4 °C with the primary anti-Caveolin-1 antibody (1:200 in blocking buffer; BD biosciences, cat. no.: 610060). Following washing with blocking buffer, cells were incubated with AlexaFluor546-conjugated secondary goat anti-rabbit antibody (1:200 in blocking solution; Invitrogen cat. no.: A11035) for 2 h at 37 °C, rewashed and mounted on microscopy slides in ProLong Gold Antifade Mountant containing DAPI (ThermoFisher; cat. no.: P36931).

Cells were imaged with Nikon Instruments Eclipse Ni-E with a C2+ confocal laser scanner (100× Plan Apo λ oil immersion objective, NA = 1.45). DAPI, GFP and AlexaFluor546 were visualized using excitation lasers of 405, 488 and 561 nm and emission filters of 445/50, 525/50 and 660 nm LP, respectively. Image acquisition was performed with NIS-Elements AR (Nikon Instruments, version 4.20). Cells were defined as regions of interest according to their EGFP expression. Subsequently, mean fluorescence intensities of GFP and CAV1 signals were measured. Analyses were performed with Fiji^37^.

### Induction of apoptosis

Transfected HEK-293T cells were kept in the dark or were exposed to blue light 5 h before treatment with 1 µM 4-Hydroxytamoxifen (4-OHT, Sigma, cat. no.: H7904) to induce apoptosis. Images were acquired 48 hours after induction with 4-OHT using a Zeiss Axio Observer D1 inverted microscope with AxioCam MRm and a 20x objective.

### Caspase8 activity assay

Transfected HEK-293T cells were kept in the dark or were exposed to blue light 3 h before treatment with 1 µM 4-OHT. All cells were detached 3 hours after induction with 4-OHT and centrifuged at 500 *g* for 10 min. The supernatant was discarded and cells washed with PBS. After centrifugation, cell pellet was resuspended in cold Lysis Buffer (10 mM Tris-Cl at pH 7.4, 100 mM NaCl, 2.5 mM MgCl_2_, 0.5% NP-40, 0.5% Triton X-100) and incubated for 10 min. The cell lysate was centrifuged at 10,000 × g for 3 min. In order to quantify the Caspase8 activity, 80 µl of the resulting supernatant were mixed with 80 µl of the Caspase-Glo® 8 reagent (Promega) in 96 well flat bottom white microplates (LumitracTM 200, Greiner) for 20 min. Luminescence intensity was measured in RLU (relative luminescence units) with the BMG FLUOstar OPTIMA Microplate Reader.

### Statistical analysis

One-way ANOVA with Tukey Pairwise Comparisons were performed using Minitab 17 Statistical Software (2010). Unpaired *t*-tests were performed using GraphPad Prism 6. Outliers for statistical analysis of CAV1 intensities were determined and excluded as described in^38^.

## Electronic supplementary material

Supplementary Information
