# Supplementary material for: Dual-controlled optogenetic system for the rapid down-regulation of protein levels in mammalian cells
Source: Sci Rep. 2018 Oct 9;8:15024. doi: 10.1038/s41598-018-32929-7 (PMC6177421; doi:10.1038/s41598-018-32929-7)
Supplement: Supplementary file 1 — Supplementary Information [file 41598_2018_32929_MOESM1_ESM.docx]

**Supplementary Information**

**Dual-controlled optogenetic system for the rapid down-regulation of protein levels in mammalian cells**

**Julia Baaske^1,2#^, Patrick Gonschorek^1,2,8,#^, Raphael Engesser^2,3^, Alazne Dominguez-Monedero^4^, Katrin Raute^1,2,5^, Patrick Fischbach^6^, Konrad Müller^1,9^, Elise Cachat^4^, Wolfgang W.A. Schamel^1,2,7^, Susana Minguet^1,2,7^, Jamie A. Davies^4^, Jens Timmer^2,3^, Wilfried Weber^1,2^* and Matias D. Zurbriggen^6^***

^1^ Faculty of Biology, University of Freiburg, Freiburg, Germany

^2^ BIOSS - Centre for Biological Signalling Studies, University of Freiburg, Freiburg, Germany

^3^ Institute of Physics, University of Freiburg, Freiburg, Germany

^4^ Deanery of Biomedical Sciences, University of Edinburgh, Edinburgh, UK

^5^ SGBM - Spemann Graduate School of Biology and Medicine, University of Freiburg, Freiburg, Germany

^6^ Institute of Synthetic Biology, University of Düsseldorf and CEPLAS, Düsseldorf, Germany

^7^ Center for Chronic Immunodeficiency, Medical Center, University of Freiburg, Faculty of Medicine, University of Freiburg, Freiburg, Germany.

* Corresponding author: Email: [wilfried.weber@biologie.uni-freiburg.de](mailto:wilfried.weber@biologie.uni-freiburg.de) or [matias.zurbriggen@uni-duesseldorf.de](mailto:matias.zurbriggen@uni-duesseldorf.de)

^#^ Julia Baaske and Patrick Gonschorek contributed equally to this work

^8^ Present address: Institute of Chemical Sciences and Engineering, School of Basic Sciences, Ecole Polytechnique Fédérale de Lausanne (EPFL), Lausanne CH-1015, Switzerland.

^9^ Present address: Novartis Pharma AG, Basel CH-4002, Switzerland

**Supplementary Information**

Development and calibration of the mathematical model

Supplementary Table S1. Fitted parameter values obtained by the maximum likelihood estimation

Supplementary Table S2. Plasmids designed and used in this study

Supplementary Table S3. Oligonucleotides designed and used in this study

Supplement Figure S1. Testing of engineered EL222-KRAB/promoter modules

Supplement Figure S2. Statistical quantification of CAV1 down-regulation

Supplement Figure S3. Multiple optimization runs with random initial parameter guesses

Supplement Figure S4. Profile likelihood of the estimated parameters

Supplement Figure S5. Prediction profile likelihood for the measured points in Figure 4

**Development and calibration of the mathematical model**

In the following the mathematical model is derived and the calibration of the unknown model parameters by maximum likelihood estimation is described.

**1. Derivation of the model equations**

In order to quantitatively characterize the output of the blue OFF system we developed a mathematical model based on ordinary differential equations (ODE) describing the time evolution of the concentrations of the involved substances. Our system can be described as a biochemical reaction network. In the following we use mass action kinetics and enzyme kinetics to derive the model equations.

The system depends on the light induced conformational change of two proteins. On the one hand the protein degradation module B-LID is fused to FLuc. The resulting FLuc-B-LID is present in two conformations: FLuc_off_ and FLuc_on_. FLuc_off_ is the present form in the dark with an inactive B-LID domain. It is translated from FLuc_mRNA_ and degrades linearly with a constant rate. Under illumination with 460nm light FLuc_off_ changes its conformation to FLuc_on_ and is available for active degradation via the proteasome. The light induced conformational change is reversed with a constant dark reversion rate. FLuc is under the control of the P_SV40_ promoter, which produces FLuc_mRNA_ at a constant rate. The FLuc protein is translated from the FLuc_mRNA_ in the OFF state. This leads to the following scheme:

Proteasome

k_on_ I(t)

k_off_

k_deg,ind_

k_translation_

k_transcription_

k_deg,mRNA_

∅

FLuc_on_

FLuc_mRNA_

k_deg,const_

k_deg,const_

∅

FLuc_off_

The time dependent intensity of the light is denoted with I(t). The second light-controlled process is a conformational change of the repressor KRAB-EL222. Activated KRAB-EL222 is able to bind to the (C120)_5_  sequence and thus inhibiting the transcription of the FLuc_mRNA_.

k_on_ I(t)

k_off_

k_transcription_

KRAB_off_

KRAB_on_

FLuc_mRNA_

The light induced conformational changes and the dark revision rates are chosen to be the same for both processes since both use the light-oxygen-voltage (LOV) domain for sensing 460 nm light. The total amount of EL222-KRAB is assumed to be in steady state since it is produced and degraded with a constant rate.

When translating these processes into mathematical equations using mass action kinetics and enzyme kinetics one obtains the following system of coupled and nonlinear ODEs:

1. $\frac{d\left[ FLuc_{off} \right]\left( t \right)}{dt}=-k_{deg,const}\left[ FLuc_{off} \right]+k_{translate}\left[ FLuc_{mRNA} \right]-k_{on}I\left( t \right)\left[ FLuc_{off} \right]+k_{off}[FLuc_{on}]$
2. $\frac{d\left[ FLuc_{on} \right]\left( t \right)}{dt}=-k_{deg,const}\left[ FLuc_{on} \right]-\frac{k_{deg,ind}\left[ FLuc_{on} \right]}{K_{m,deg}+\left[ FLuc_{on} \right]}+k_{on}I(t)\left[ FLuc_{off} \right]-k_{off}\left[ FLuc_{on} \right]$
3. $\frac{d\left[ KRAB_{off} \right]\left( t \right)}{dt}=-k_{on}I\left( t \right)\left[ KRAB_{off} \right]+k_{off}[KRAB_{on}]$
4. $\frac{d\left[ KRAB_{on} \right]\left( t \right)}{dt}=+k_{on}I\left( t \right)\left[ KRAB_{off} \right]-k_{off}[KRAB_{on}]$
5. $\frac{d\left[ FLuc_{mRNA} \right]\left( t \right)}{dt}=\frac{k_{transcript}}{1+k_{inh,KRAB}\left[ KRAB_{on} \right]^{2}}-k_{deg,mRNA} [FLuc_{mRNA}]$

The induced degradation via the proteasome in equation (2) is modelled with saturating Michaelis-Menten kinetics. The maximal degradation rate is k_deg,ind_. For FLuc_on_ = K_m,deg_ the half of the maximal degradation rate is reached. The repressor KRAB has multiple binding sites on the DNA. To capture cooperative binding effects we included an exponent of two in equation (5).

**2. Estimation of the unknown model parameter by fitting to experimental data**

2.1. Maximum likelihood approach

The mathematical model is calibrated with experimental data. To estimate the unknown model parameters, we used an approach based on the maximum likelihood. The equations (1)-(5) can be written in a vectorized form:

1. $\frac{d}{dt}\boldsymbol{x}\left( t \right)=\boldsymbol{f}(\boldsymbol{x,p,u}\left( t \right))$

The vector with the internal states $\boldsymbol{x}\left( t \right)\epsilon\mathbb{R}^{n}$ contains the concentrations of the involved substances at the time point *t*. The function ***f*** is describing the reaction kinetics. The reactions are depending on model parameters ***p*** which are typically unknown. Additionaly, the system depends on a time dependent external input, i.e. the light intensity *I(t)*, that is described by the function ***u***(t). To obtain a unique solution of the system of ODEs one has to define an initial state $\boldsymbol{x}_{0}=\boldsymbol{x}(0)$.

Usually the concentrations $\boldsymbol{x}(t)$ of the involved proteins cannot be measured directly. To map the internal states $\boldsymbol{x}\left( t \right)$ to the experimentally accessible observables $\boldsymbol{y}(t)$ we define the observation function $\boldsymbol{g}$:

1. $y\left( t \right)=\boldsymbol{g}\left( \boldsymbol{x}\left( t \right),\boldsymbol{s} \right)+\boldsymbol{\varepsilon}(t)$

In our system, the observation parameters ***s*** are scaling parameters. $\boldsymbol{\varepsilon}(t)$ models the measurement error. A common error distribution for concentration measurements is the log-normal distribution^[1]^. The logarithm $\log\left( y_{i} \right)$ of log-normal distributed data $y_{i}$ is normal distributed

1. $\log\left( y_{i} \right)\sim N(\mu_{j},\sigma^{2})$

As error model, we assume the same variance $\sigma^{2}$ for all measured data points, which is estimated simultaneously with the dynamic parameters from the experimental data.

Assuming one single experiment with only one observable we can now calculate the probability of the measured data set $\boldsymbol{y}^{D}$ given a parameter vector $\theta=(\boldsymbol{p,}\boldsymbol{x}_{\boldsymbol{0}}\boldsymbol{,s},\sigma)$

1. $L\left( \boldsymbol{y}^{D},\boldsymbol{\theta} \right)= \prod_{j=i}^{N_{D}} \exp\left( \frac{\left( \log\left( y_{j}^{D} \right)-\log\left( g\left( \boldsymbol{x}\left( t_{j} \right),\boldsymbol{s} \right) \right) \right)^{2}}{2\sigma^{2}} \right)$

*N_D_* is the number of data points and *t_j_* are the time points of measurement. When having multiple experiments with multiple observables the product of the single probabilities is giving the overall probability of the measured data

1. $L\left( \boldsymbol{y}^{D},\boldsymbol{\theta} \right)=\prod_{i} L_{i}(\boldsymbol{y}_{i}^{D},\boldsymbol{\theta})$

$L(\boldsymbol{y}^{D}, \boldsymbol{\theta})$ seen as function of $\boldsymbol{\theta}$ for a given data set $\boldsymbol{y}^{D}$ is called likelihood function. With this, one can define the maximum likelihood estimator $\hat{\boldsymbol{\theta}}$ of the parameter set $\boldsymbol{\theta}$

1. $\hat{\theta}=\underset{\theta}{\arg\max} (L\left( \boldsymbol{y}^{D},\boldsymbol{\theta} \right))$

Instead of maximizing the likelihood *L* it is numerically advantageous to minimize

1. $-2logL=\sum_{j=i}^{N^{D}} \left( \frac{\log\left( y_{J}^{D} \right)-\log\left( g\left( \boldsymbol{x}\left( t_{j} \right),\boldsymbol{s} \right) \right)}{\sigma} \right)^{2}+2N_{D}\log\left( \sqrt{2\pi}\sigma\right)=:\chi_{mod}^{2}(\boldsymbol{\theta})$

This is just the sum of the weighted squared residuals in logarithmic space $\chi^{2}\left( \theta\right)=\sum_{j=i}^{N^{D}} \left( \frac{\log\left( y_{J}^{D} \right)-\log\left( g\left( \boldsymbol{x}\left( t_{j} \right),\boldsymbol{s} \right) \right)}{\sigma} \right)$ with an additional term due to the error model. The optimal parameter set is then obtained by taking the minimum

1. $\hat{\boldsymbol{\theta}}=\underset{\theta}{arg\min} (\chi_{mod}^{2}(\boldsymbol{\theta}))$

To assess parameter uncertainties in terms of confidence intervals one can calculate the profile likelihood^[2]^ for each parameter $\theta_{i}$

1. $\chi_{PL}^{2}\left( \theta_{i} \right)=\min_{\theta_{i\neq j}} \chi_{mod}^{2}(\theta)$

The 95 % confidence interval can be calculated with

1. $CI\left( \theta_{i} \right)=\left\{ \boldsymbol{\theta}|\chi_{mod}^{2}(\boldsymbol{\theta}) \right.-\chi_{mod}^{2}\left( \hat{\boldsymbol{\theta}} \right)<\chi^{2}(95\%,df=1)\}$

where $\chi^{2}\left( 95\%,df=1 \right)$ denotes the 95 %-quantile of a $\chi^{2}$-distribution with one degree of freedom.

2.2. Scaling invariances and initial concentrations

In the following we describe the two experiments used for the calibration of the model and derive the used initial conditions.

Since EL222-KRAB is not measured, the absolute concentration of EL222-KRAB is not accessible by the model. Scaling the concentration of EL222-KRAB by a factor α can be compensated by transforming the parameters

*k_inh,KRAB_* *k_inh,KRAB_* ⋅ α^-2^

init_KRAB_off_ init_KRAB_off_ ⋅ α

init_KRAB_on_ init_KRAB_on_ ⋅ α.

The observations ***y****(t)* are invariant under these transformations. Since α is arbitrary we can set *α*= init_KRAB^-1^_off_ and therefore init_KRAB_off_ = 1. The concentration of EL222-KRAB is then measured in multiples of the initial EL222-KRAB concentration in the OFF state.

The same argumentation is possible for the FLuc_mRNA_ therefore we can set init_FLuc_mRNA_ = 1. In both experiments the cells were cultivated for 16 hours in the dark, therefore we assume that all KRAB and FLuc is in the OFF state at the start of the actual experiment. This leads to the initial concentrations:

FLuc_off_(0) = init_FLuc

FLuc_on_(0) = 0

KRAB_off_(0) = 1

KRAB_on_(0) = 0

FLuc_mRNA_(0) = 1

2.3. Implementation of the single experiments

***Experiment 1: Kinetics***

In this experiment the cells were cultivated for 16 hours in the dark and then illuminated with blue light with the intensity I(t) = 20 μmol m^-2^ s^-1^. At 0, 2, 4 and 8 h after illumination the amount of FLuc was measured. As observation function, we used:

FLuc_obs(t) = [FLuc_on_](t) + [FLuc_off_](t)

without a scaling factor. This means the concentration scale of FLuc is determined by this experiment.

The time course was measured for four different conditions:

1. with the full dual-controlled system,
2. without light responsive KRAB,
3. without light responsive FLuc,
4. without any light responsive module.

Condition (i) is the full system as described in equation (1)-(5). To implement the conditions (ii)-(iv) following parameter transformations were used

1. k_inh,KRAB_ = 0,
2. k_deg,ind_ = 0,
3. k_inh,KRAB_ = 0 and k_deg,ind_ = 0.

***Experiment 2: Light intensity dose response***

In this experiment the system was incubated for 16 hours in the dark and then illuminated with different light intensities for 8 hours. The system was simulated for 8 hours for the different in the experiment applied light intensities. As observation function we used

FLuc_obs(8 h) = scale_DR_ ⋅ ([FLuc_on_](8 h) + [FLuc_off_](8 h))

The experimental conditions were the same as in Experiment 1.

2.4. Fitting results

In total twelve parameters were fitted to the experimental data shown in Figure 3. The fitting and uncertainty analysis was performed with the Data2Dynamics framework^[3]^.

The ODEs were simulated numerically with the CVODES integrator of the SUNDIALS suite^[4]^. As optimization algorithm to minimize $\chi_{mod}^{2}(\boldsymbol{\theta})$ we used a trust region algorithm implemented in the MATLAB function *lsqnonlin* with user supplied sensitivities^[5]^.

The parameters were fitted on a logarithmic scale, this improves convergence since the parameter space is scanned over orders of magnitude and only positive parameter values are possible. To find the global optimum we performed 1000 optimization runs with randomly sampled initial parameter guesses. More than 98 % of these converged to the same lowest minimum (Supplementary Figure S3). This is a very strong indication that the global optimum was found. The parameter values of the best fit are shown in Supplementary Table S1, the corresponding model curves describing the fitted data are shown in Figure 3. The shaded error bands are showing one standard deviation of the estimated error model, which assumes a normal error on the logarithmic scale.

The calculated likelihood profiles are shown in Supplementary Figure S4. The corresponding 95 % parameter confidence intervals are denoted in Supplementary Table S1. All parameters expect of k_on_ and k_off_ are identifiable. The light induced confirmation change k_on_ and the dark revision rate k_off_ are practical non-identifiable since the profile likelihood flattens out for high parameter values. This means, it is not possible to estimate an upper bound for the parameter values and the confirmation change can be arbitrarily fast. This result is in agreement with biological knowledge, since a confirmation change of a protein can happen within seconds and therefore on a much faster timescale than other cellular processes like gene expression (hours) or proteasome dependent protein degradation (minutes to hours). The profile likelihood can also be utilized to identify model reductions in order to obtain a fully identifiable model^[6]^. The analysis reveals that the ratio K=k_off_/k_on_ is identifiable. Hence, the model can be reduced by applying a steady state approximation for FLuc-B-LID and KRAB-EL222.

1. $\left[ FLuc_{on} \right]\left( t \right)=I\left( t \right)K[FLuc_{off}](t)$
2. $\left[ {KRAB}_{on} \right]\left( t \right)=I\left( t \right)K[{KRAB}_{off}](t)$

With this reduction the model becomes fully identifiable by the experimental data.

**3. Model based description of the characteristics of the systems**

With the calibrated mathematical model, it is possible to predict the performance of the system for different light intensities and durations of illumination. The heatmap in Figure 4a was obtained by simulating the calibrated system for the indicated light intensities and illumination durations and plotting the resulting reporter gene expression. This prediction can be seen as characterization of the system since it is possible to find experimental conditions to obtain a desired target gene expression.

To test the predictive power of the model, we measured the expression level of the reporter gene FLuc for different combinations of illumination duration and light intensity. Uncertainties in the parameter estimation are leading to uncertainties of the predictions. These prediction uncertainties can be calculated by evaluating the prediction profile likelihood for each prediction^[7]^. The resulting prediction profiles are shown in Supplementary Figure S5. The calculation was done with the doPPL plugin for the Data2dynamics software^[8]^.

**Supplementary Tables**

**Supplementary Table S1.** Fitted parameter values obtained by the maximum likelihood estimation. σ^–^ and σ^+^ are indicating the 95 % confidence interval obtained by the profile likelihood analysis. The corresponding likelihood profiles are shown in Supplementary Figure S4.

| **Parameter** | **θ_opt_** | **σ ^-^** | **σ^+^** | **Unit** |
| --- | --- | --- | --- | --- |
| k_deg,ind_ | 1.226 | 0.8867 | 1.777 | h^-1^ ⋅ RRE |
| k_deg,const_ | 0.4248 | 0.2748 | 0.6692 | h^-1^ |
| K_m,deg_ | 0.1301 | 0.02259 | 0.2665 | RRE |
| k_on_ | 1.883 | 0.1286 | +inf | h^-1^ ⋅ (μmol m^-2^ s^-1^)^-1^ |
| k_off_ | 22.75 | 0.8333 | +inf | h^-1^ |
| k_translate_ | 1.132 | 0.7497 | 1.724 | h^-1^ ⋅ RRE ⋅ [FLuc_mRNA_](0)^-1^ |
| k_transcript_ | 0.5428 | 0.3000 | 1.093 | h^-1^ ⋅ [FLuc_mRNA_](0) |
| k_inh,KRAB_ | 4.424 | 2.254 | 11.61 | [KRAB_off_](0)^-2^ |
| k_deg,mRNA_ | 0.2539 | 0.1117 | 0.5233 | h^-1^ |
| init_FLuc | 1.010 | 0.9324 | 1.093 | RRE |
| scale_DR_ | 0.2196 | 0.2001 | 0.2410 | 1 |
| sd_FLuc_ | 0.07217 | 0.06493 | 0.08086 | 1 |

* RRE = relative reporter expression

**Supplementary Table S2.** Plasmids designed and used in this study.

If not indicated otherwise, all plasmids were cloned using AQUA^[9]^/ Gibson Cloning^[10]^.

| **Plasmid** | **Description** | Reference or source |
| --- | --- | --- |
| pGL4.23-C120-FLuc | Vector encoding FLuc under the control of a minimal promoter with inserted C120. | ^[11]^ |
| pJB010 | **P_CMV_-CAV1-B-LID- IRES-GFP-pA**  B-LID was amplified from pMZ1203 using oligos oMZ1214F/ oJB3-005R. The backbone pRR-CMV-CAV1-IRES-GFP was amplified in two fragments using primer pairs AmpF/ oJB3-006R and oJB3-007F/ AmpR. All 3 fragments were assembled using Gibson Cloning. | **this work** |
| pJB013 | **P_CMV_-KRAB-EL222- IRES-GFP-pA**  KRAB-EL222 was amplified using oJB3-008F/ oJB3-009R from pKM565. The backbone pRR-CMV-CAV1-IRES-GFP was amplified without CAV1 in two fragments using primer pairs AmpF/ oJB3-010R and oJB3-011F/ AmpR. All 3 fragments were assembled using Gibson Cloning. | **this work** |
| pJB023 | **P_CMV_-(C120)_5_-CAV1-B-LID-IRES-GFP-pA**  (C120)_5_ was amplified from pMZ1203 using oJB-064F/ oJB-058R. CAV1-B-LID was amplified from pJB010 using oJB-060F/ oJB-063R. Both fragments were fused in a PCR reaction using oligos oJB-064F/oJB-063R. The backbone pRR-CMV-CAV1-IRES-GFP was digested using BamHI/ XbaI. The C120-CAV1-B-LID fragment was assembled into digested backbone using Gibson Cloning. | **this work** |
| pJB036 | **P_SV40_-2x(C120)_5_-FLuc-B-LID-pA**  Amplifiy (C120)_5_ sequence from pMZ1203 with additional SacI restriction site in overhang using oJB103/oJB104. Digest pMZ1203 and PCR product with SacI and ligate fragments. | **this work** |
| pJB037 | **P_CMVtrunc_-(C120)_5_-FLuc-B-LID-pA**  Amplify pMZ1203 with SV40 sequence using AmpF/oJB097 and oJB096/AmpR. Further amplify PCR product of oJB096/AmpR with oJB098/AmpR to completely add CMVtrunc sequence (^[12]^). Assemble fragments using Gibson cloning. | **this work** |
| pJB038 | **P_PGK_-(C120)_5_-FLuc-B-LID-pA**  Amplify PGK promoter sequence from pMSCVneo using oJB099/oJB100. Amplify pMZ1203 without promoter sequence using AmpF/oJB101 and pJB102/AmpR. Assemble all fragments with Gibson cloning | **this work** |
| pJB039 | **P_SV40_-2x(C120)_5_-FLuc-B-LID_ΔRRRG_-pA**  Amplify pJB036 using AmpF/oMZ1253R and oMZ1252F/AmpR. Assemble fragments with Gibson cloning. | **this work** |
| pJB040 | **P_CMVtrunc_-(C120)_5_-FLuc-B-LID_ΔRRRG_-pA**  Amplify pJB037 using AmpF/oMZ1253R and oMZ1252F/AmpR. Assemble fragments with Gibson cloning. | **this work** |
| pJB041 | **P_PKG_-(C120)_5_-FLuc-B-LID_ΔRRRG_-pA**  Amplify pJB038 using AmpF/oMZ1253R and oMZ1252F/AmpR. Assemble fragments with Gibson cloning. | **this work** |
| pKM528 | **P_EF1α_-NLS-EGFP-Med25VBD-B-LID-pA**  Vector encoding PEF1a-controlled nuclear-localized EGFP-Med25VBD-B-LID | ^[13]^ |
| pKM565 | **P_SV40_-KRAB-EL222-pA**  KRAB was amplified from pWW43 using oligos oKM455/oKM456, digested (NheI/EcoRI) and ligated (NheI/EcoRI) into pVP-EL222. | **this work** |
| pMSCVneo | Retroviral cloning vector containing PGK promoter. | Clontech |
| pMZ1203 | **P_SV40_-(C120)_5_-FLuc-B-LID-pA**  C120-FLuc was amplified from pGL4.23-C120-FLuc (oMZ1212F/oMZ1213R), B-LID was amplified from pKM528 (oMZ1214F/oMZ1215R) and both fragments were assembled into NotI and XbaI digested pMZ333. | **this work** |
| pMZ1210 | **P_SV40_-C120-FLuc-B-LID_ΔRRRG_-pA**  Whole pMZ1203 except of the four amino acids RRRG was PCR amplified in two fragements using the two primer pairs (AmpF/oMZ1253R & oMZ1252F/AmpR) and assembled again. | **this work** |
| pMZ333 | PSV40 driven mammalian expression vector derived from XbaI/NotI digested pSAM200 (^[14]^) . | ^[15]^ |
| pMZ-BLID-mCherry-2A-myrcasp8-ER(T2) | BLID-mCherry-2A-Myrcasp8-ER(T2) produced as gblocks (IDT) and inserted in pMZ1203 via gibson assembly | **this work** |
| pRR-CMV-CAV1-IRES-GFP | **P_CMV_-CAV1-IRES-EGFP** | ^[16]^ |
| pTREX-BLID-mCherry-2A-myrcasp8-ER(T2) | BLID-mCherry-2A-myrcasp8-ER(T2) was PCR amplified  (primers: pMZ-BLID F and pMZ-BLID R) and cloned into a pTREX backbone (primers: pTREX F and pTREX R) via gibson assembly | **this work** |
| pTREX-myrCasp8-ER(T2)-IRES-mCherry | myristoylated caspase 8 fused to the mutant ligand binding domain of the estrogen receptor (primers: attB1-myrCasp8 and attB2-ER(T2)) inserted into pTREx-DEST-IRES-mCherry through Gateway® recombination | ^[17]^ |
| pVP-EL222 | **P_SV40_-NLS-VP16-EL222-pA** | ^[11]^ |
| pWW43 | **P_SV40_-E-KRAB-pA** | ^[18]^ |

**Supplementary Table S3.** Oligonucleotides designed and used in this study.

| **Primer** | **Sequence** |
| --- | --- |
| AmpF | 5’ - GCTCCTTCGGTCCTCCGATCG - 3’ |
| AmpR | 5’ - ACTTCTGACAACGATCGGAGGACC - 3’ |
| oJB-058R | 5’ - CCCTCGGAGTCTACGTATTTGCCCCCAGACATGGTGGCTTTACCAACAGTACCG- 3’ |
| oJB-060F | 5’ - ATGTCTGGGGGCAAATACGTAG- 3’ |
| oJB-063R | 5’ - GAATTCGAAGCTTGAGCTCGAGGCCTGCAGGGATCCTTAGCCGCGGCGGCGGGCGGCCTCGTCGATG- 3’ |
| oJB-064F | 5’ - GCTGTTTTGACCTCCATAGAAGACACCGACTCTAGAAGCTACCTGAGCTCGCTAGCCTCGAG- 3’ |
| oJB-096 | 5’ - CATTAGTTCATAGCCCATATATGGAGTTCCGCGTTACATAACTTACGGTAAATGGCCCGCCTGCCCTCG  AGGAACTGGAAAACC- 3’ |
| oJB-097 | 5’ - ATTACTATTAATAACTAGTCAATAATCAATGTAGCTAGCTTATCGATGATAAGCTGTC- 3’ |
| oJB-098 | 5’ –ACATTGATTATTGACTAGTTATTAATAGTAATCAATTACGGGGTCATTAGTTCATAGCCCATATATGGAG  TTC-3’ |
| oJB-099 | 5’ - TGTTTGACAGCTTATCATCGATAAGCTAGCTTGGGTAGGGGAGGCGCTTTTCC- 3’ |
| oJB-100 | 5’ - GTTAACTTTCTGGTTTTCCAGTTCCTCGAGGGCGAAAGGCCCGGAGATGAGGAAG- 3’ |
| oJB-101 | 5’ - AGCTAGCTTATCGATGATAAGCTGTC- 3’ |
| oJB-102 | 5’ - CCCTCGAGGAACTGGAAAACC- 3’ |
| oJB-103 | 5’ - GATCGAATTGCGGCCGC- 3’ |
| oJB-104 | 5’ - ATCGAGGAGCTCCTTCCATTATATACCCTCTAGTGTCTAAGC- 3’ |
| oJB3-005R | 5’ - TTAGCCGCGGCGGCGGGCGGCCTCGTCGATG - 3’ |
| oJB3-006R | 5’ - GTAGTAGCCAAAAAGGCGCCGGCGCCGCTAGCTATCTCTTTCTGCGTGCTGATGC - 3’ |
| oJB3-007F | 5’ - CATCGACGAGGCCGCCCGCCGCCGCGGCTAAGGATCCCTGCAGGCCTC - 3’ |
| oJB3-008F | 5’ - CTCCATAGAAGACACCGACTCTAGAAGCATGGATCCAAAAAAGAAGAGAAAGGTAGATCC - 3’ |
| oJB3-009R | 5’ - GCTTGAGCTCGAGGCCTGCAGGGATCCTCAGATTCCGGCTTCGACGGC - 3’ |
| oJB3-010R | 5’ - CATGCTTCTAGAGTCGGTGTCTTC - 3’ |
| oJB3-011F | 5’ - TGAGGATCCCTGCAGGCC - 3’ |
| oKM455 | 5’ - TCCAGGCACATGCGTCCGCGTGCTAGCCCCACCATGGATCCAAAAAAGAAGAGAAAGGTAGATC - 3’ |
| oKM456 | 5’ - caagtcGAATTCCCAGAGATCATTCCTTGCCATTC - 3’ |
| oMZ1212F | 5’ - TGTCTTTTATTTCAGGTCCCGGATCGAATTGCGGCCGCTACCTGAGCTCGCTAGCCTCGAG - 3’ |
| oMZ1213R | 5’ - GTAGTAGCCAAAAAGGCGCCGGCGCCGCTAGCCACGGCGATCTTGCCGCC - 3’ |
| oMZ1214F | 5’ - GCTAGCGGCGCCGGCGCC - 3’ |
| oMZ1215R | 5’ - GGATCGAAGCTTGGGCTGCAGGTCGACTCTAGATTAGCCGCGGCGGCGG - 3’ |
| oMZ1252F | 5’ - CATCGACGAGGCCGCCTAATCTAGAGTCGACCTGCAGCCC - 3’ |
| oMZ1253R | 5’ - GGTCGACTCTAGATTAGGCGGCCTCGTCGATGTTC - 3’ |
| pTREX F | 5’ - GCAGCCCAAGCTTCTACCCAGCTTTCTTGTACAAAGTGGTTGATGG- 3’ |
| pTREX R | 5’ - TTCCAGTTCCTCGAGGGAAGCCTGCTTTTTTGTACAAACTTGTTGATGG- 3’ |
| pMZ-BLID F | 5’ - TACAAAAAAGCAGGCTTCCCTCGAGGAACTGGAAAACC- 3’ |
| pMZ-BLID R | 5’ - GCTGGGTAGAAGCTTGGGCTGCAGGTCGACTCTA- 3’ |
| attB1-myrCasp8- | 5’ - GGGGACAAGTTTGTACAAAAAAGCAGGCTTCACCATGGGGAGTAGCAAGAGC- 3’ |
| attB2-ER(T2) | 5’ - GGGGACCACTTTGTACAAGAAAGCTGGGTCTAAGCTGTGGCAGGGAAACC- 3’ |


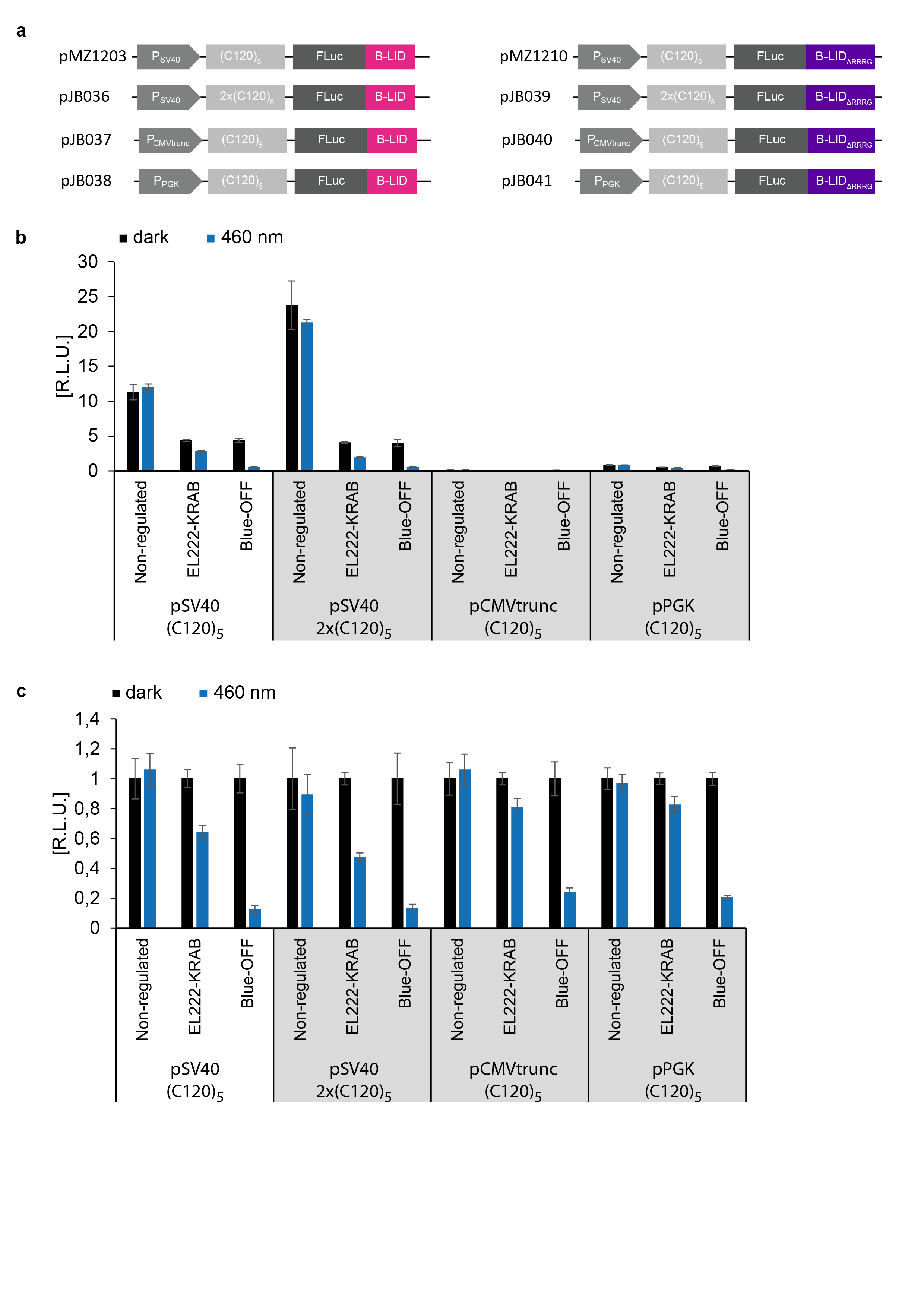


**Supplement Figure S1.** Evaluation of repression efficiency of a set of engineered EL222-KRAB/promoter modules as single and dual regulation systems (Blue-OFF). **(a)** Constructs of different variants of the promoter module of the reporter plasmids: i) system displayed in the main text containing a single EL222 binding sequence, (C120)_5_ (pMZ1203 and pMZ1210) or ii) two repeats thereof, 2x(C120)_5_, downstream of a P_SV40_ promoter (pJB036 and pJB039); and two different promoter versions: iii) a truncated version of the pCMV, pCMVtrunc (pJB037 and pJB040), and iv) the phosphoglycerate kinase promoter, pPGK (pJB38 and pJB041). **(b)** and **(c)** Cells were transfected with KRAB-EL222 (pKM565) and the respective reporter plasmids, kept for 16 hours in darkness after transfection and subsequently placed in darkness or 20 µmol m^-2^ s^-1^ 460 nm light for 8 h before lysis. **(b)** Firefly luciferase luminescence normalized to Renilla luciferase luminescence (relative luminescence units, R.L.U.). The ‘non-regulated’ samples have no blue light responsive repression or degradation module, i.e. pWW43 with either pMZ1210/pJB039/pJB040/pJB041. ‘EL222-KRAB’ depicts the EL222-KRAB module as single regulation system (pKM565 with either pMZ1210/pJB039/pJB040/pJB041), ‘Blue-OFF’ describes the complete dual system with both light-responsive modules: pKM565 (blue-light repression, EL222-KRAB) and either pMZ1203/pJB036/pJB037/pJB038 (blue-light degradation, B-LID). **(c)** blue light data from (b) normalized to dark controls. None of the engineered variants (grey shade) based on either two repeats of the EL222 binding site, a truncated version of the pCMV or the pPGK promoters showed improved performance in comparison to the promoter P_SV40_-(C120)_5_ which was used throughout the work.


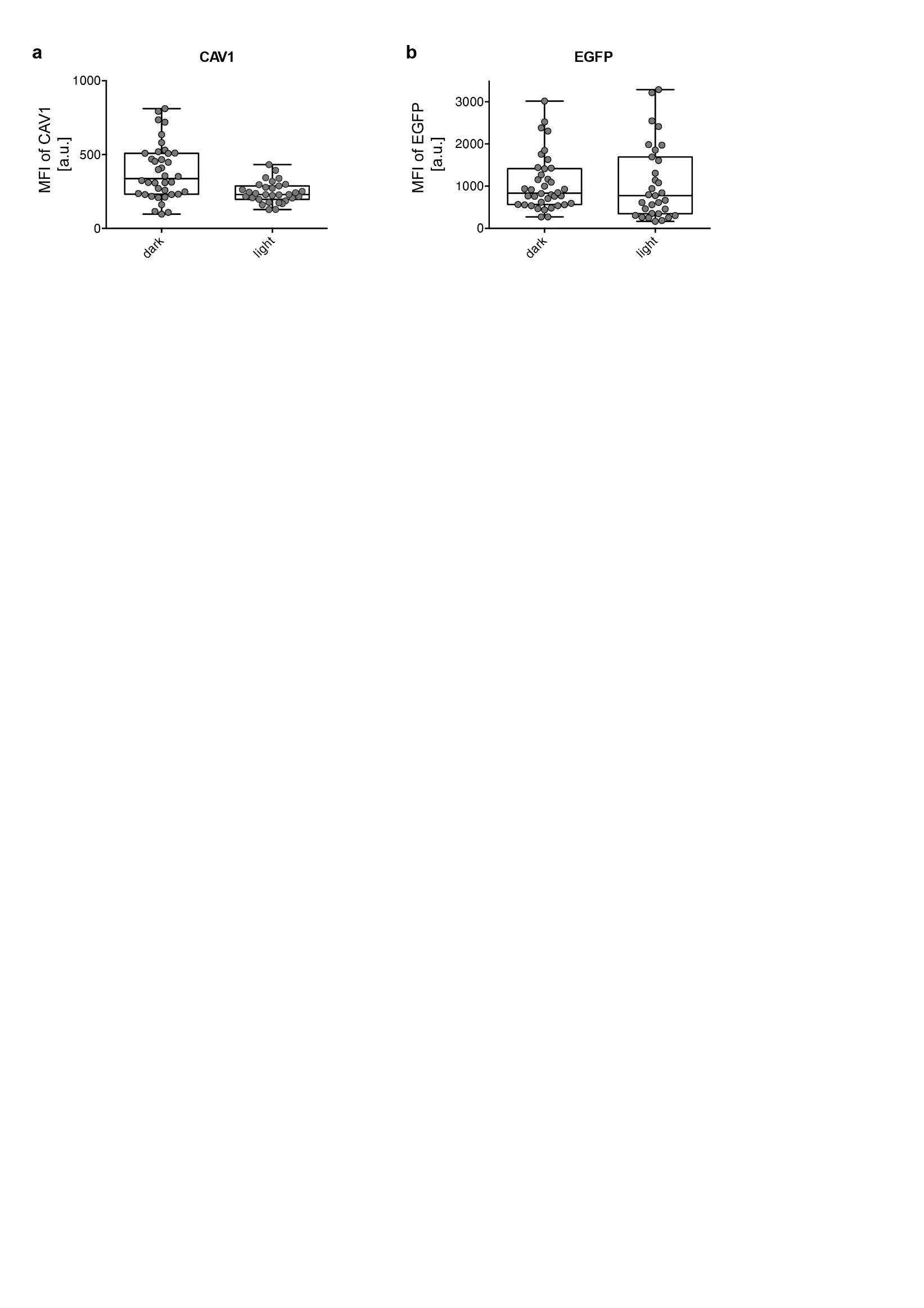


**Supplement Figure S2.** Statistical quantification of CAV1 downregulation in MEF cells **(a)** Mean fluorescent intensities (MFI) of CAV1 staining of transfected cells after 16 h in darkness (n= 38) or under 2 µmol m^-2^ s^-1^ of 460 nm light (n=31). Unpaired t-test shows significant difference (P = 0.0001) **(b)** Mean fluorescent intensities of GFP co-expression in analysed cells (P = 0.4752)

**Supplement Figure S3.** Multiple optimization runs with random initial parameter guesses sorted by their –2 log(L) value. The steps are indicating local minima. More than 98 % converged to the same optimum.

**Supplement Figure S4.** Profile likelihood of the estimated parameters. The solid lines indicate the profile likelihood; the optimal parameter set is marked with a grey star. The red dashed line marks the 95 % confidence level. The parameter axis is on a logarithmic scale.

**Supplement Figure S5.** Prediction profile likelihood for the measured points in Figure 4. The solid lines indicate the prediction profile likelihood, the prediction for the optimal parameter set is marked with a grey star. The red dashed line marks the 95 % confidence level.

**Supplementary References**

[1] C. Kreutz, M. M. Bartolome Rodriguez, T. Maiwald, M. Seidl, H. E. Blum, L. Mohr, J. Timmer, *Bioinformatics* **2007**, *23*, 2747–2753.

[2] A. Raue, C. Kreutz, T. Maiwald, J. Bachmann, M. Schilling, U. Klingm??ller, J. Timmer, *Bioinformatics* **2009**, *25*, 1923–1929.

[3] A. Raue, B. Steiert, M. Schelker, C. Kreutz, T. Maiwald, H. Hass, J. Vanlier, C. Tönsing, L. Adlung, R. Engesser, et al., *Bioinformatics* **2015**, *31*, 3558–3560.

[4] A. C. Hindmarsh, P. N. Brown, K. E. Grant, S. L. Lee, R. Serban, D. E. Shumaker, C. S. Woodward, *ACM Trans. Math. Softw.* **2005**, *31*, 363–396.

[5] T. Coleman, Y. Li, *SIAM J. Optim.* **1996**, *6*, 418–445.

[6] T. Maiwald, H. Hass 1☯, B. Steiert 1☯, J. Vanlier, R. Engesser, A. Raue, F. Kipkeew, H. H. Bock, D. Kaschek, C. Kreutz, et al., **n.d.**, DOI 10.1371/journal.pone.0162366.

[7] C. Kreutz, A. Raue, J. Timmer, *BMC Syst. Biol.* **2012**, *6*, 120.

[8] H. Hass, C. Kreutz, J. Timmer, D. Kaschek, *Bioinformatics* **2016**, *32*, 1204–1210.

[9] H. M. Beyer, P. Gonschorek, S. L. Samodelov, M. Meier, W. Weber, M. D. Zurbriggen, *PLoS One* **2015**, *10*, e0137652.

[10] D. G. Gibson, L. Young, R.-Y. Chuang, J. C. Venter, C. A. Hutchison, H. O. Smith, C. A. H. Iii, N. America, *Nat. Methods* **2009**, *6*, 343–345.

[11] L. B. Motta-Mena, A. Reade, M. J. Mallory, S. Glantz, O. D. Weiner, K. W. Lynch, K. H. Gardner, *Nat. Chem. Biol.* **2014**, *10*, 196–202.

[12] S. Kainrath, M. Stadler, E. Reichhart, M. Distel, H. Janovjak, *Angew. Chemie Int. Ed.* **2017**, *56*, 4608–4611.

[13] K. Müller, M. D. Zurbriggen, W. Weber, *Biotechnol. Bioeng.* **2015**, *112*, 1483–1487.

[14] M. Fussenegger, S. Moser, X. Mazur, J. E. Bailey, *Biotechnol. Prog.* **1997**, *13*, 733–740.

[15] S. Juillot, H. M. Beyer, J. Madl, W. Weber, M. D. Zurbriggen, W. Römer, *Mol. BioSyst.* **2016**, *12*, 345–349.

[16] R. Strippoli, J. Loureiro, V. Moreno, I. Benedicto, M. L. Pérez Lozano, O. Barreiro, T. Pellinen, S. Minguet, M. Foronda, M. T. Osteso, et al., *EMBO Mol. Med.* **2014**, *7*, 102–123.

[17] E. Cachat, W. Liu, J. A. Davies, *Eng. Biol.* **2017**, 1–6.

[18] W. Weber, C. Fux, M. Daoud-el Baba, B. Keller, C. C. Weber, B. P. Kramer, C. Heinzen, D. Aubel, J. E. Bailey, M. Fussenegger, *Nat. Biotechnol.* **2002**, *20*, 901–907.
